# Antimicrobial Resistance in the Food Chain: A Review

**DOI:** 10.3390/ijerph10072643

**Published:** 2013-06-28

**Authors:** Claire Verraes, Sigrid Van Boxstael, Eva Van Meervenne, Els Van Coillie, Patrick Butaye, Boudewijn Catry, Marie-Athénaïs de Schaetzen, Xavier Van Huffel, Hein Imberechts, Katelijne Dierick, George Daube, Claude Saegerman, Jan De Block, Jeroen Dewulf, Lieve Herman

**Affiliations:** 1 Directorate Control Policy, Federal Agency for the Safety of the Food Chain (FASFC), Kruidtuinlaan 55, Brussels 1000, Belgium; E-Mail: xavier.vanhuffel@favv.be; 2 Faculty of Bioscience Engineering, Ghent University, Coupure Links 653, Ghent 9000, Belgium; E-Mails: sigrid.vanboxstael@ugent.be (S.V.B.); eva.vanmeervenne@ugent.be (E.V.M.); 3 Institute of Agricultural and Fisheries Research, Brusselsesteenweg 370, Melle 9090, Belgium; E-Mails: els.vancoillie@ilvo.vlaanderen.be (E.V.C.); jan.deblock@ilvo.vlaanderen.be (J.D.B.); lieve.herman@ilvo.vlaanderen.be (L.H.); 4 CODA-CERVA, Veterinary and Agrochemical Research centre, Groeselenberg 99, Brussels 1180, Belgium; E-Mails: patrick.butaye@coda-cerva.be (P.B.); hein.imberechts@coda-cerva.be (H.I.); 5 Faculty of Veterinary Medicine, Ghent University, Salisburylaan 133, Merelbeke 9820, Belgium; E-Mail: jeroen.dewulf@ugent.be; 6 Scientific Institute of Public Health, Juliette Wytsmanstraat 14, Brussels 1050, Belgium; E-Mails: boudewijn.catry@wiv-isp.be (B.C.); katelijne.dierick@wiv-isp.be (K.D.); 7 Faculty of Veterinary Medicine, University of Liège, Boulevard de Colonster 20, Liège 4000, Belgium; E-Mails: marie-athenais.deschaetzen@provincedeliege.be (M-A.S.); george.daube@ulg.ac.be (G.D.); claude.saegerman@ulg.ac.be (C.S.); 8 Scientific Committee of the FASFC, Kruidtuinlaan 55, Brussels 1000, Belgium

**Keywords:** antimicrobial resistant bacteria, antimicrobial resistance genes, horizontal gene transfer, food safety

## Abstract

Antimicrobial resistant zoonotic pathogens present on food constitute a direct risk to public health. Antimicrobial resistance genes in commensal or pathogenic strains form an indirect risk to public health, as they increase the gene pool from which pathogenic bacteria can pick up resistance traits. Food can be contaminated with antimicrobial resistant bacteria and/or antimicrobial resistance genes in several ways. A first way is the presence of antibiotic resistant bacteria on food selected by the use of antibiotics during agricultural production. A second route is the possible presence of resistance genes in bacteria that are intentionally added during the processing of food (starter cultures, probiotics, bioconserving microorganisms and bacteriophages). A last way is through cross-contamination with antimicrobial resistant bacteria during food processing. Raw food products can be consumed without having undergone prior processing or preservation and therefore hold a substantial risk for transfer of antimicrobial resistance to humans, as the eventually present resistant bacteria are not killed. As a consequence, transfer of antimicrobial resistance genes between bacteria after ingestion by humans may occur. Under minimal processing or preservation treatment conditions, sublethally damaged or stressed cells can be maintained in the food, inducing antimicrobial resistance build-up and enhancing the risk of resistance transfer. Food processes that kill bacteria in food products, decrease the risk of transmission of antimicrobial resistance.

## 1. Introduction

The availability of antibiotics for treating infectious diseases significantly improved the health and life expectancy of humans as well as the health and welfare of animals. However, the use of antibiotics results in a selection for antimicrobial resistance in bacteria. Antimicrobial resistance is a worldwide problem for both public and animal health. Food may act as a vector for the transfer of antimicrobial resistant bacteria and antimicrobial resistance genes to humans.

Various scientific studies support the hypothesis of a link between the use of antibiotics during agricultural production and antimicrobial resistance of human pathogens in which food is one of the possible transfer routes [1,2,3,4,5,6,7,8]. The large majority of the antibiotics are used in primary animal production. For example, pork and poultry meat can both be sources of transfer of antimicrobial resistant *Salmonella* Typhimurium strains to humans [2,7]. A recent study estimated the probability of exposure to 1,000 colony forming units of cephalosporin resistant *Escherichia coli* (CREC) through consumption of a meal containing chicken meat as ca. 1.5% [2]. Aquaculture is also of concern and studies have shown an important antimicrobial use [9] and resistance concern [10]. In primary plant production (horticulture) and apiculture (bees), the use of antibiotics is assumed to be low. The streptomycin preparation Fructocin, for example, is a plant protection product which is forbidden since 25 December 2002 for the treatment of fire blight (*Erwinia amylovora*) in apple and pear trees [11].

This review focuses on the impact of food processing on the transfer of antimicrobial resistance to humans.

## 2. Antimicrobial Resistance

### 2.1. Definition

In general, antimicrobial resistance is the capacity of a microorganism to resist the growth inhibitory or killing activity of an antimicrobial beyond the normal susceptibility of the specific bacterial species [12,13,14]. Antimicrobials comprise any substance that has a growth inhibiting of killing effect on microorganisms in a clinical setting or for reducing bacterial loads in materials and surfaces. They include antibiotics, which are used to treat bacterial infections in humans and animals, as well as chemical biocides, which are used for disinfection in the food processing environment (see Section 4.3). A microorganism can acquire resistance to an antimicrobial to which it was previously sensitive, meaning that the antimicrobial will no longer be able to kill or inhibit the growth of the microorganism at the same level as before. Three types of resistance are described. Microbiological resistance (*in vitro* resistance) means a reduced susceptibility of bacteria to antibiotics above a breakpoint that is defined by the upper limit of normal susceptibility of the concerned species, which is also called epidemiological resistance. The microbiological resistance can often be confirmed genotypically by demonstrating the presence of a certain antimicrobial resistance gene or resistance mechanism via molecular techniques. Secondly, there is the pharmacological resistance. This is based on pharmacokinetic parameters and the normal susceptibility of a bacterial species. If the minimal inhibitory concentration (MIC) of the antibiotic for the bacteria concerned is within the concentration range that can be attained by that antimicrobial, it is susceptible. If the MIC of the antibiotic for the concerned bacteria is higher than the concentration that can be attained at the site of infection, then the bacterium is regarded as resistant. Finally, clinical resistance (*in vivo* resistance) means an infection with the concerned bacterium cannot be treated appropriately anymore and treatment failures are evident [15].

Scientific publications reveal that antimicrobial resistance is sometimes accompanied by a lower bacterial biological fitness. This has been demonstrated for *Streptococcus pneumoniae* with resistance to macrolides [16] and for *Acinetobacter* sp. with resistance to rifampicin [17]. Whereas for other bacteria and resistance types, antimicrobial resistance in pneumococci is accompanied by unchanged or increased biological fitness when compared to sensitive pneumococci [18].

### 2.2. Mechanisms of Antimicrobial Resistance

Bacteria can be resistant to antibiotics by using several mechanisms: enzymatic degradation of antibiotics, antibiotic target modification, changing the bacterial cell wall permeability and alternative pathways to escape the activity.

Enzymatic degradation or modification of antibiotics is a very common mechanism of resistance. Examples are the β-lactamase enzymes hydrolyzing the β-lactam ring of β-lactam antibiotics such as the cephalosporins, which are mainly of concern in Gram-negative bacteria [19]. Another group of antibiotics to whom resistance is mainly mediated by enzymatic degradation are the aminoglycosides, where inactivation is caused by acetyltransferases, nucleotidyltransferases and phosphotransferases [20]. Each of these enzymes exists in many variants having each a specific spectrum for one or more antibiotics.

Resistance by target modification implies a modification of the target molecule of the antibiotic, in general an enzyme, so that the antibiotic loses its binding capacity and hence its activity. Examples of this mechanism are mutations in the gyrase and topoisomerase genes that are the targets of the quinolone and fluoroquinolone antibiotics [21]. Methicillin resistant *Staphylococcus aureus* (MRSA) are an example of horizontal transmissible target modification. MRSA contain the *mecA* gene coding for a variant penicillin binding protein PBP2A having a very low affinity for β-lactams. In the presence of β-lactams, the only PBP that remains functional is the low affinity PBP2A [22].

Changing the cell wall or cell envelope permeability implies reducing entry or increasing the efflux of antibiotics, thereby regulating the internal concentration of antibiotics in the cell. Changes in pores can alter or inhibit the entering capability of antibiotics into the cell. Efflux can be increased specifically by acquisition of specific genes, as exemplified by tetracycline resistance [23]. On the other hand, increased efflux can be due to the over-expression of physiologically present efflux pumps, causing in general a multidrug resistant phenotype. This mechanism is not transferred among bacteria. The levels of resistance caused by these pumps are generally discrete and clinical relevance remains unclear.

Finally, cells can become resistant by deviating from their normal physiological pathway by including an alternative step. In general this is caused by an extra enzyme. This is exemplified by the production of an additional dihydrofolate reductase, with an R-plasmid-determined trimethoprim resistance, which differs from the chromosomal enzyme in its binding to various anti-folate compounds in *Escherichia coli* and *Citrobacter* sp. [24].

Antimicrobial resistance can be intrinsic or acquired. Intrinsic antimicrobial resistance is an inherent characteristic of a bacterial species or genus towards a certain antibiotic. As a consequence, treatment with this antibiotic will not be successful [25]. It might even worsen a certain clinical condition because of triggering subinfection by other intrinsic resistant pathogens, like e.g., *Clostridium difficile* [26] in man or *Trueperella* (*Arcanobacterium*)* pyogenes* in bovines [27]. Antimicrobial resistance is acquired when a susceptible strain has become resistant as a consequence of a recent evolution of the strain. This can be the result of either a mutation, which is generally a spontaneous event happening within a bacterial population, or the acquisition of a specific resistance gene by horizontal gene transfer (HGT).

### 2.3. Mechanisms of Horizontal Gene Transfer

**Figure 1:**
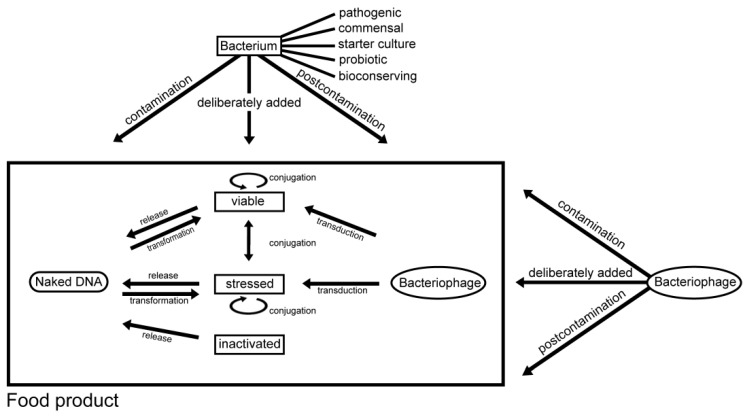
Overview of horizontal gene transfer in food products.

There are three main mechanisms of HGT between bacteria: conjugation, transformation and transduction. These may occur in the soil, in water, in the digestive system of humans and animals, as well as in food. Figure 1 depicts the HGT in food products. HGT of antimicrobial resistance genes, their maintenance in bacterial populations and the creation of multidrug resistance is greatly enhanced by genetic structures such as plasmids, integrons and transposons [25,28,29,30]. These are mobile genetic elements since they represent a pool of mobile DNA. The frequency of HGT largely depends on the properties of the mobile genetic element, the characteristics of the donor and recipient populations, and the environment.

In addition to conjugation, transduction and transformation, other less well recognized mechanisms of DNA transfer may occur in nature [31]. These include vesicle-mediated translocation of antimicrobial resistance and virulence genes that originate from one cell fused with another by a range of Gram-negative bacteria, transfer by virus-like particles and mixing of entire genomes by cellular fusion occurring in multicellular bacteria.

#### 2.3.1. Conjugation

Conjugation is the transfer of DNA that occurs between live bacterial cells and requires direct contact between the donor and the recipient cell. Antimicrobial resistance genes are very often present on mobile elements such as plasmids and transposons, and can be supplementary associated with insertion elements, integrons and genomic islands. Transposons and insertion sequences translocate within bacterial cells. Plasmids and other mobile genetic elements may result in complex assemblies. Conjugation involves the transfer of conjugative or non-conjugative plasmids or transposons, the latter recently classified as Integrative Conjugative Elements (ICE) [32,33,34] or Integrative Mobilizable Elements (IME) [35]. ICEs and IMEs also contain genomic islands. Transfer of a non-conjugative plasmid is possible when other mobile elements offer the necessary *tra* genes in trans. Such plasmid fusion is often facilitated by the presence of insertion elements of transposons on the plasmid(s). Mobile genetic elements cause a certain genomic plasticity, however, they are frequently themselves plastic too. Complex integrations of transposons, integrons and insertion sequences into ICEs and IMEs have been described on more than one occasion [36,37]. An example of plasticity is the IME, named *Salmonella* genomic island 1 (SGI1), found in several *Salmonella* serovars and in *Proteus mirablis*. The originally found SGI1 carried resistance genes to ampicillin, chloramphenicol, florfenicol, streptomycin, spectinomycin, sulfonamides and tetracycline. Actually, numerous variants have been described [37,38,39]. It has even been shown that during a ten year period, in a same *Salmonella* Agona clone, only the SGI1 changes while the genetic background of the strain remains the same [40]. An integron itself is an immobile element which can capture, integrate and express or release gene cassettes. They were first described in the late 1980s [41] and two groups can be distinguished, the mobile integrons (MI), associated with mobile DNA elements as transposons and plasmids, and the chromosomal integrons (CI) associated with the bacterial chromosome [42]. The MIs can be divided into five different integron classes, but only the first three classes are historically associated with the dissemination of multiresistance [42]. Several studies indicate a clear link between integrons and multiresistance. For instance, for verotoxin producing *Escherichia coli* (VTEC), Nagachinta and Chen [43] reported that all integron positive strains examined were resistant to at least three different antibiotics. Van Meervenne *et al.* [44] found that 91.3% of the integron positive VTEC strains were resistant to at least three different antibiotics.

Because many antimicrobial resistance genes have been found on mobile elements like plasmids and transposons, conjugation is considered as the main mode of antimicrobial resistance gene transfer among bacteria [13]. However, conjugation is limited by a number of molecular and epidemiological factors. First, the ecosystem should allow contact between the strains. Next to that, the strains should have a certain mobility, whether by themselves or by external factors. Third, there is incompatibility between plasmids avoiding the transfer of plasmids with the same incompatibility group entering a same cell. Fourth, and this is more for IMEs, the genetic background of the cell should allow the integration of the IME. Finally, some mobile genetic elements have maintenance systems that when they are expelled of the cell, the cell can die.

Bacteria have developed different systems for plasmid transfer, but some basic conjugative steps can be found in all these systems. In Gram-negative bacteria, conjugation seems to follow a general mechanism starting with the formation of conjugative pili to mediate contact between donor and recipient cells. Gram-positive bacteria use alternative mechanisms to achieve cell contact, such as e.g., pheromone-induced plasmid transfer in enterococci [45] or aggregation-mediated plasmid transfer in *Bacillus thuringiensis* subsp. *israelensis* [46].

#### 2.3.2. Transformation

Transformation of bacteria is the process where naked DNA from the environment is taken up in bacterial cells [47]. Transformation includes the following steps. Bacterial DNA is first released from bacterial cells either passively after death and lysis or, for some bacteria, actively at a specific point in the growth cycle [48,49]. The DNA is then taken up by competent bacteria in the vicinity. Next to that, the DNA survives the destructive nucleases in the bacterial cell and is stable incorporated in the acceptor cell. Finally, the incorporated DNA is expressed.

Theoretically any bacterial chromosomal or extra-chromosomal DNA can be transferred by transformation. Several bacterial species are naturally competent (e.g., *Campylobacter* spp., *Bacillus subtilis* and especially *Streptococcus* spp.) [50]. The time when competence is induced seems species-dependent. Some naturally transformable species are competent throughout the logarithmic growth phase, e.g., *Acinetobacter* spp., while others, such as *Streptococcus pneumoniae*, are competent for only short time periods, or as *Bacillus subtilis*, which develops competence only at the onset of the stationary phase [51]. On the other hand, competence can also be constitutively expressed, as is the case in *Neisseria gonorrhoeae* [51]. Some species like *Neisseria gonorrhoeae* and *Haemophilus influenzae* are selective and present a sequence specific uptake system [48]. Due to the differences in their cell wall structure, differences can be found in the DNA uptake systems of Gram-positive and Gram-negative bacteria, although they use similar proteins [52].

Competence in some bacterial species such as *E. coli* can be induced *in vitro* by chemical or physical conditions such as the presence of CaCl_2_, EDTA, temperature shifts, electro-shocks or lightening [53,54,55]. It can be hypothesized that several minimal processing methods likewise could induce bacterial competence.

Till now, the significance of antimicrobial resistance gene transfer in food products by transformation has not been shown. This is probably due to the lower frequency and difficulty to detect the event compared to conjugation. This lower frequency is the consequence of the different requirements that have to be fulfilled before it results in a successful transfer of antimicrobial resistance genes. In the human and animal gut and during food processing, DNA is vulnerable to the action of DNA nucleases, physical degradation (e.g., by heat, shearing forces) and chemical degradation processes. This was shown in fruit juice [56] and in a variety of different other food products [57]. However, the complex food matrix and the food processing environment (e.g., biofilms) can protect the DNA as is shown in sausages [58]. DNA can also be protected by individual food components (e.g., arginine, maltol) [57,59]. The stability of DNA during food processing is an inverse function of the DNA length [60,61]. In order to be stabilized in the recipient cell, the transformed DNA must be available as a plasmid or must recombine with homologous regions in the resident chromosome. Absence of homologous sequences or origins of replication were identified as major barriers to HGT by transformation [31,62,63,64].

#### 2.3.3. Transduction

Transduction is a bacteriophage-mediated transfer process. First, the bacteriophage attaches to the bacterium and injects its genetic material, potentially including host bacterial DNA. After entering the bacterial cell, the DNA has to be stabilized either by forming an autonomously replicating element or by integration in the bacterial DNA. Once the foreign DNA is stabilized in the bacterial cell, it can direct the production of new phage particles. In this way, bacterial plasmid and/or genomic DNA of different lengths can be transferred from one bacterium to another, depending on the phage involved.

The host range of this mechanism may be rather limited due to the host specificity of bacteriophages and therefore, transduction occurs in general between closely related bacterial strains. However, the transducing capacity of a phage is not necessary limited to bacteria which can be infected but can be wider [65,66].

Till now, transfer of antimicrobial resistance genes by transduction is only rarely reported. For *Staphylococcus aureus*, transfer of the plasmid-borne *qacB* gene, coding for a multidrug efflux protein, and transfer of antimicrobial resistance plasmids by transduction have been reported [67,68].

## 3. Antimicrobial Resistance in Food

### 3.1. Contamination of Food with Antimicrobial Resistant Bacteria and Antimicrobial Resistance Genes

Food may be contaminated with antimicrobial resistant bacteria and/or antimicrobial resistance genes in many ways. Antimicrobial resistant bacteria may be found in the soil, in the water and in human or animal fecal material. Animal products may contain antimicrobial resistant bacteria as a result of fecal contamination during slaughter. Plant products may be contaminated with antimicrobial resistant bacteria during production following the use of irrigation water contaminated with human and/or animal feces or by sewage discharges [69]. Food may also be contaminated by the environment. Such contamination may occur after food processing and is then referred to as post-contamination. Finally, food can be contaminated with antimicrobial resistant bacteria and/or antimicrobial resistance genes originating from other foods during the handling of the food by the consumer. This is called cross-contamination.

Conjugation in food matrices was reported in experimental studies e.g., the transfer of plasmid-borne ampicillin resistance genes from *Salmonella* Typhimurium to *E. coli* K12 in inoculated sterilized milk and ground beef [70] and the transfer of antimicrobial resistance from lactic acid bacteria (LAB) (*Enterococcus faecalis*, *Lactococcus lactis*) to potential pathogenic strains (*Listeria* spp., *Salmonella* spp., *Staphylococcus aureus* and *E. coli*) in fermented whole milk (fermented with the LAB donors) [71]. Van der Auwera *et al.* [72] found significant levels of conjugation and mobilization of plasmids between strains of *Bacillus thuringiensis* in milk and rice pudding. The highest transfer frequencies were obtained in milk, in which conjugative transfer was approximately ten-fold higher compared to liquid LB medium. Also, conjugation has been suggested from epidemiological data as shown by the presence of the same conjugative plasmids and integrons in bacteria isolated from romaine lettuce, savoy spinach and alfalfa sprouts [73].

In food products, the development of competence for uptake of DNA and transformation has been shown for *Bacillus subtilis* in milk [74,75]. Transformation has been supposed to be an important mechanism for the antimicrobial resistance transfer to *Campylobacter jejuni*. Cj1211, an inner membrane transporter protein involved in the transfer of DNA across the membrane, is a key player in the natural transformation of *Campylobacter jejuni* [76]. However, capsular polysaccharide and lipooligosaccharide have been shown to restrict this transformation process [77].

### 3.2. Intentional Addition of Microorganisms (with Antimicrobial Resistance Properties) to Food as Auxiliary Technical Substances

During the production process of certain food products, microorganisms which can contain antimicrobial resistance genes, are intentionally added for technical reasons. According to the intended effect, these microorganisms can be classified into four groups: starter cultures, probiotics, biopreserving microorganisms and bacteriophages.

Starter cultures are microbiological cultures that are added to foodstuffs in order to induce the onset of fermentation. LAB are mostly used for that purpose (*Lactobacillus*, *Lactococcus*, *Leuconostoc* and *Pediococcus*). Some starter cultures may also have probiotic properties or be used for biopreservation. Starter cultures are used in fermented food and drinks, e.g., yoghurt, fermented sausages and sauerkraut.

Probiotics are live microorganisms that are added to foodstuffs for their positive effects on the host organism. LAB and bifidobacteria are the most common types of microorganisms used as probiotics. Certain yeasts (e.g., *Saccharomyces boulardii*) and bacilli are also used for that purpose. Probiotics are mainly added to fermented foods such as yoghurt and supplementary food. They are also used as feed supplements.

Biopreservation is the use of natural or controlled microbiota as a way of extending the shelf life of food. Such bacteria may inhibit or inactivate spoilage organisms and pathogens because they compete for nutrients and/or produce antimicrobial agents. Moreover, they may also have fermenting or probiotic properties. LAB may be used for the purpose of biopreservation of various foodstuffs, including fermented food and boiled meat products. Because they produce acids and bacteriocins, they have an antibacterial action against spoilage organisms and pathogenic bacteria such as *Listeria monocytogenes* [78,79,80]. Some LAB of the genus *Enterococcus* have an inhibiting effect on the most relevant spoilage organisms in fish and crustaceans and may therefore be added to such foods [81]. The yeast *Pichia anomala* may be added to plant products such as cereals because of its antifungal effect as well as its inhibiting effect on Gram-negative bacteria, including *Enterobacteriaceae* [82,83,84]. *Lactococcus plantarum* and *Lactococcus pentosus* may be used for the biopreservation of fish (bass) because of their antagonistic activity against psychrotrophic, pathogenic and coliform bacteria [85].

A general finding on antimicrobial resistance in starter cultures is that transferable resistance genes are rare and that resistance against tetracycline is most common [86]. Antimicrobial resistance is sometimes detected in fermented food [87] and in probiotic strains [88]. Among the LAB isolated from spontaneously fermented foodstuffs, resistance is most common in *Enterococcus*. In most cases, this bacterium is resistant to vancomycin although resistances to tetracycline, erythromycin and chloramphenicol have also been observed [87]. *Enterococcus*, *Lactococcus* and *Lactobacillus* containing multiresistant plasmids have already been isolated from dairy products [87,89]. Among *Lactobacillus* isolated from artisan cheese, a high incidence of tetracycline and erythromycin resistance has been detected [90]. Tetracycline resistance occurs rather often in LAB associated with raw meat [91]. A German study showed that six out of the 473 examined probiotic LAB isolated from human and animal isolates, were multiresistant to tetracyline and erythromycin [92]. In *Lactococcus* and *Streptococcus thermophilus* isolated from dairy products a high incidence of resistance to tetracycline en erythromycin has been found [93]. Resistance to tetracycline has also been detected in probiotic bifidobacteria, including seven *Bifidobacterium animalis* subsp. *lactis* and *Bifidobacterium bifidum* strains [88]. Katla *et al.* [94] found one *Lactobacillus* strain out of 189 isolates of LAB, analyzed for their sensitivity to fourteen antibiotics, that was resistant to streptomycin. In a Swiss study, resistance to tetracycline was found in *Staphylococcus* isolates used as starter cultures in meat as well as in *Bifidobacterium lactis* and in *Lactobacillus reuteri.* Resistance to lincosamide was detected in *Lactobacillus reuteri* [95]. Resch *et al.* [96] found antimicrobial resistance in coagulase-negative staphylococci isolated from cheese, sausages and meat. It was remarkable to see that all staphylococci were sensitive to the clinically significant antibiotics.

Bacteriophages are host specific viruses of bacteria (phagetypes) and may therefore be used to inactivate foodborne pathogens and spoilage organisms. Scientific literature contains descriptions of the control of *Listeria monocytogenes* on soft cheese [97] and on honeydew melon [98,99] and of *Campylobacter jejuni* [100,101] and *Salmonella enteritidis* [101] on chicken skin by bacteriophages.

The prevalence of antimicrobial resistance genes in bacteriophages is largely unknown. Three genes conferring resistance to β-lactam-antibiotics, *i.e.*, two β-lactamase-genes (*bla*_TEM_ and *bla*_CTX-M9_) and one gene coding for a modified penicillin-binding protein (*mecA*) were found in all 30 bacteriophage DNA samples from urban waste water and river water. This study shows that bacteriophages could be environmental reservoirs of antimicrobial resistance genes [102]. These genes were also found in nearly all the bacteriophage DNA samples from fecal waste of pigs, poultry and cattle. This study shows that bacteriophages might act as environmental factors for the horizontal transfer of antimicrobial resistance genes [103].

Since starter cultures, probiotics and biopreserving microorganisms often comprise the same bacterial genera, the transfer of antimicrobial resistance occurs via the same mechanisms. After ingestion of food, the added microorganisms end up in the human digestive system where the transfer of bacterial genes may take place. In most cases, transfer occurs via conjugation, although theoretically transformation and transduction cannot be ruled out.

*In vitro* research showed the transfer of antimicrobial resistance genes by means of conjugation of LAB to Gram-positive and Gram-negative bacteria [13], and among LAB [71], e.g., of tetracycline resistance genes from *Lactobacillus plantarum* to *Lactococcus lactis* and *Enterococcus faecalis* [104], of erythromycin resistance genes from *Lactobacillus fermentum* and *Lactobacillus salivarius*, and of tetracycline resistance genes from *Lactobacillus plantarum* and *Lactobacillus brevis* to *Enterococcus faecalis* [105], from *Lactobacillus curvatus* and *Enterococcus faecalis* to *Lactobacillus curvatus* [106], and of tetracycline and erythromycin resistance genes among *Enterococcus faecalis* isolates [107].

The transfer of antimicrobial resistance genes by conjugation has also been demonstrated in food, namely the transfer of tetracycline resistance genes among LAB in fermented milk [71], among *Lactobacillus curvatus* in fermented sausages [106], and of tetracycline and vancomycin resistance genes among *Enterococcus faecalis* during the fermentation process of cheese and sausages [107].

As outlined before, bacteriophages are host specific and it is assumed that transduction by phages only occurs between closely related strains, mostly belonging to one single species. However, transduction of a pathogenicity island by phages between *Staphylococcus aureus* and *Listeria monocytogenes* has already been demonstrated [108]. So far, the transfer of antimicrobial resistance by means of transduction has rarely been reported for *in vitro* studies. Scientific literature describes the transfer of genes coding for the multidrug efflux proteins *qacA* and *qacB* in MRSA strains by means of transduction [67]. To the best of our knowledge, transfer of antimicrobial resistance by means of transduction on food products has not yet been documented, despite the presence of resistance genes in phages from *Salmonella* and VTEC strains [109].

Most added LAB microorganisms colonize the intestines during a short period of time. Probiotics, however, have the ability to attach to the intestinal epithelial cells. This ability is strain-specific. As a result, probiotics are able to colonize the intestines for a longer period of time, thus increasing the risk of transfer of antimicrobial resistance genes, when compared to short-term colonizing strains. Moreover, foods containing probiotics are consumed on a large scale [110].

Microorganisms of which large numbers are present in a foodstuff or in human intestines are more likely to transfer antimicrobial resistance genes than microorganisms of which only small numbers are present. This is the case for probiotics and biopreserving microorganisms when large amounts are added to foodstuffs, and for starter cultures which grow during the fermentation process.

The risk of starter cultures being resistant is believed to be higher in the case of spontaneous fermentation than in the case of a fermentation involving the use of starter cultures because starter cultures strains may be checked for the presence of transferable antimicrobial resistance genes. In the EU this risk is currently very low for bacteriophages since no bacteriophages have yet been approved for use in foodstuffs. In the USA, bacteriophages have already been commercialized, e.g., LISTEX^TM^ for *Listeria monocytogenes* in cheese and ListShield^TM^ for *Listeria monocytogenes* in food products [111].

It is necessary to make sure that microorganisms intentionally added to food are free from transferable antimicrobial resistance genes. In the USA, microorganisms that are added in order to ferment food are evaluated on the basis of the food grade or Generally Recognized As Safe (GRAS) principle [112]. In Europe, this evaluation is made on the basis of the Qualified Presumption of Safety (QPS) concept, including a list of microorganisms generally considered safe for use. The QPS status is granted to a particular taxonomic group of microorganisms on the basis of the determination of the identity, the amount of available knowledge, the possible pathogenicity and the final use. Further safety analyses are no longer required for microorganisms belonging to a QPS group. As for microorganisms that are added to foodstuffs, Regulation (EC) No. 258/97 on novel foods should be applied [113]. The use of bacteriophages as food additives is forbidden, as up till now, no application has been authorized within the EU.

## 4. Transfer of Antimicrobial Resistance in the Food Processing Environment

### 4.1. Influence of Food Processing and Preservation Techniques

Food processing and preservation techniques are applied in order to extend the shelf life of foodstuffs. They can have different effects on their bacterial flora. Bacteria present in food products can survive after the application of a food processing or preservation technique, without their growth being inhibited. It is also possible that their growth is inhibited, resulting in stressed or sublethally damaged bacterial cells. Finally, food processing and/or preservation techniques can kill or inactivate the bacteria. Those dead bacterial cells can stay intact or can be lysed due to cell wall damage. As a consequence, the bacterial DNA, including the eventual present antimicrobial resistance genes, are liberated in the environment. Most food processing methods result in a reduction of the bacterial count [114].

Raw food products may be ingested without any prior processing or preservation treatment (e.g., fresh vegetables and fruit, raw milk) and may contain live, non-stressed bacterial cells at the time of ingestion. Hence, such foodstuffs may hold a high risk for the transfer of antimicrobial resistance since possibly present antimicrobial resistant bacteria are not killed. Transfer of antimicrobial resistance genes from live bacteria to other bacteria in the foodstuff or in the intestines after ingestion by humans may occur by means of conjugation. There is an increasing demand for raw and minimally processed food. These markets expand because they combine an optimal taste with a maximum preservation of nutritional components.

In the food processing industry, minimal processing technologies or preservation treatments are applied to obtain a safe and stable product [115]. Examples of such processes are: cooling, acidification, modified atmosphere packaging, freezing, mild pasteurization, intense pulsed light treatment and UV radiation treatment [116,117,118,119]. Dependent on the combination (multiple hurdle technology), type and conditions of the applied technologies, these techniques may result in the control of growth, a decrease of the microbial load but also in stressed and or sublethally damaged cells [120].

Stress conditions such as cold stress, heat stress, acid stress, freeze injury among others may trigger several mechanisms in bacterial cells, e.g., stress adaptation, cellular repair, application of response mechanisms and enhanced virulence [121]. But besides these mechanisms, several studies demonstrated that stress may also impact the phenotypic antimicrobial resistance of the microorganisms.

McMahon *et al.* [122] for example have shown that sublethal food preservation stresses such as heat stress, acid and salt stress can significantly alter phenotypic antimicrobial resistance in food-related pathogens such as *E. coli*, *Salmonella* Typhimurium and *Staphylococcus aureus*. On one hand, sublethal high temperature decreased antimicrobial resistance, while increased salt or reduced pH conditions on the other hand increased the phenotypical antimicrobial resistance. A similar observation on salt stress was made by Ganjian *et al.* [123] who found that *Staphylococcus aureus* showed increased phenotypic antimicrobial resistance after being exposed to sublethal concentrations of salt.

Al-Nabulsi *et al.* [124] compared the phenotypic antimicrobial resistance for 13 antimicrobials of *Cronobacter sakazakii* isolated from infant powder before and after exposure to sublethal stress treatments such as cold stress, heat stress, acid stress and alkaline stress. They found for several stress/antimicrobial combinations an impact depending on the combination. For example for cold stress, strains were more sensitive to 4 from the 13 tested antimicrobials, but more sensitive to the other antimicrobials. The heat stressed strains were in general more resistant than the sensitive strains.

The mechanisms lying at the basis of the increased or decreased phenotypic antimicrobial resistance is in most cases not specifically known. However, a follow-up study by McMahon *et al.* [125] on the observation of increased phenotypic resistance of *Salmonella* Typhimurium and *Escherichia coli* showed that the applied sublethal preservation conditions increase horizontal transmission by means of conjugation of plasmids containing antimicrobial resistance genes, when compared to the frequency found between non-stressed bacterial cells. They also showed that some of the pathogens continue to express higher levels of phenotypic antimicrobial resistance after removal of stress, suggesting that, in some cases, the applied sublethal stress has a residual effect on antimicrobial susceptibilities. These studies suggest that increased use of sublethal, rather than lethal food preservation systems may contribute to the development and dissemination of antimicrobial resistance in pathogens [122].

Several of the minimal processing conditions such as UV radiation but also heating may cause damage of the bacterial DNA [121]. One of the answers of the bacteria to this damage is the SOS response mechanism, which is an inducible DNA repair system [126]. Van der Veen and Abee [127] have recently reviewed this SOS response from a food safety perspective. Cirz *et al.* [128] have demonstrated a link between the activity of the SOS response after exposal to stress and increased antimicrobial resistance in pathogenic *E. coli*. Apart from the SOS response, bacteria can have several molecular response systems. Bacteria thus have various systems to counteract stress and they will use these systems in food processing environments.

Antimicrobial resistance genes that are present in partly inactivated, stressed cells may be transferred to commensals and pathogens, both in the foodstuff and after ingestion in the digestive system of humans. This may be achieved either by conjugation, when resistance is located on mobilizable elements, or by transformation and transduction, however to a lower degree.

A large number of food processing methods are applied in order to kill bacterial cells. The goal of the combination technology used in minimal processing is to expose bacteria to different hurdles, which they should not overcome. The different hurdles applied might not only have an additive effect, but can act synergistically as well [129]. Dead cells may remain intact or be lysed as a result of cell membrane damage, releasing bacterial DNA, including possible antimicrobial resistance genes, into the environment. Heat treatments such as sterilization, UHT treatment and (full) pasteurization under well defined time/temperature combinations will kill bacterial cells. Dead cells cannot pass antimicrobial resistance genes to other bacteria by conjugation or transduction. As soon as DNA has been released, antimicrobial resistance genes may, theoretically, be transferred by transformation. The process of transformation, however, occurs with a low frequency and is subject to a large number of requirements. According to the current knowledge, these bactericidal food processing methods hold the lowest risk of antimicrobial resistance transfer.

### 4.2. Influence of Biofilms

On the majority of food processing equipment, microorganisms can grow and survive as biofilms. Biofilms can be defined as a microbial derived sessile community characterized by cells that are irreversibly attached to a substratum or interface or to each other, which are embedded in a matrix of extracellular polymeric substances that they have produced, and which exhibit an altered phenotype with respect to growth rate and gene transcription [130]. Both mono- and multispecies biofilms can be found in the food industry, where they can lead to food spoilage and production of out-of-specification products. Furthermore, biofilms can also threat public health when pathogenic species are involved. Biofilms have given rise to concerns for food safety in for example the meat industry [131], the dairy industry [132] and the produce industry [133]. An important health issue related to the occurrence of biofilms in the food industry is the inherent higher antimicrobial resistance compared to planktonic cells. Several factors have been reported to contribute to this feature, such as the matrix, the growth rate, the heterogeneity within the biofilm, the general stress response and quorum sensing [134,135]. Besides this inherent resistance, the biofilm state confers an ideal state for resistance transfer and this has been shown to occur both by conjugation and transformation [136,137,138,139]. Luo *et al.* [140] studied the relationship between conjugation and biofilm development in *Lactococcus lactis*. They found that a cell-clumping-associated high-frequency conjugation system, which transmitted biofilm-forming elements among the lactococcal population, also served as an internal enhancer facilitating the dissemination of the broad-host-range drug resistance gene-encoding plasmid pAMβ1 within *L. lactis*.

### 4.3. Cross-Resistance to Antibiotics and Chemical Biocides

Bacterial cells may be exposed to chemical biocides in the food processing environment and may as a result be stressed and/or inactivated. Intrinsically, bacterial spores are the most resistant to biocides, followed by mycobacteria; Gram-negative bacteria are more sensitive and Gram-positive bacteria show the highest sensitivity to biocides [141]. Resistance to biocides depends, among others, on the presence of the bacterium in a biofilm and is generally due to a reduced permeability of the cells [141].

When bacterial cells are inactivated by biocides, their DNA may be released in the food environment as a result of lysis. Free DNA may be absorbed by bacteria through the process of transformation given conditions for successful transfer are met (see above Section 2.3.2).

As a consequence, cross-resistance to biocides and antibiotics may develop [142], and concepts of sublethal damage or stressed bacteria as outlined under minimal food processing (see Section 4.1) are of concern. Recent studies prove the existence of an epidemiological relationship between increased resistance to quaternary ammonium components in clinical *E. coli* isolates and increased resistance to cotrimoxazole and amoxicilline [143]. Another recent study shows that one single exposure to certain biocides may provoke the selection of mutant *Salmonella* Typhimurium with an efflux mediated multidrug resistance [144]. Literature studies however suggest that the relationship between antimicrobial resistance and biocide resistance does not show a consistent pattern. When *Serratia marcescens* was exposed to cetylpyridinium chloride, the strain showed an increased as well as a decreased resistance to certain biocides and antibiotics [145]. Yet, the development of cross-resistance between biocides and antibiotics depends on the nature of the biocide and the antibiotic, as well as on the circumstances. *In vitro* exposure of *Salmonella* Typhimurium to both quaternary ammonium components and triclosan provoked an increased resistance to antibiotics whereas exposure to both a mixture of oxidizing components and a disinfectant derived from phenolic tar acids did not provoke an increased resistance to antibiotics although it resulted in a higher resistance to biocides [146]. Genetic linkages between quaternary ammonium compound and β-lactam antibiotics in coagulase-negative staphylococci have been found [147,148]. Cross-resistance is to be expected particularly in the case of Gram-negative bacteria, the outermost cell layers of which acting as a barrier against antibiotics and hydrophobic molecules with a higher molecular weight [149]. Transfer of resistance that resulted from biocide treatments to other bacteria has not yet been demonstrated.

## 5. Consequences of Foodborne Antimicrobial Resistance for the Consumer

Antimicrobial resistant pathogenic bacteria may be ingested by consumers and present an immediate risk for public health. The consequences of antimicrobial resistant *Salmonella* and *Campylobacter* spp. for consumers have been studied repeatedly [150,151,152,153]. The studies revealed that the emerging resistance of these foodborne pathogens results in an increase in the number of hospitalizations and increases the risk of invasive infections and mortality.

Antimicrobial resistance genes present in foodstuffs, either contained in bacteria and bacteriophages or as DNA fragments, may involve an indirect risk for public health as they increase the gene pool from which (pathogenic) bacteria can pick up antimicrobial resistance genes and possibly transfer them to other (pathogenic) bacteria. *In vitro* studies demonstrated the transfer of erythromycin resistance genes from LAB to *Listeria* spp. [71]. The transfer of tetracycline and erythromycin resistance genes from *Enterococcus faecalis* to *Listeria monocytogenes* has been demonstrated both *in vitro* and in the digestive system of mice [154].

The first effect of antimicrobial resistant pathogenic germs is that medical treatment may fail. A second effect is that the choice of antibiotics for treatment is limited and the third effect is that resistant gastrointestinal pathogens will acquire an advantageous position when patients are treated with antibiotics for other medical reasons. Finally, antimicrobial resistance may be accompanied by a possible higher risk of increased virulence which may be due to a co-selection of resistance and virulence properties through integration of virulence and resistance plasmids [155,156]. Increased virulence may also result from an increased regulation of both virulence determinants and resistance determinants [157].

Antimicrobial resistance in commensals constitutes an indirect public health risk as antimicrobial resistance genes may be transferred to pathogens. For example, *E. coli* strains which are ingested with food may contain extended spectrum β-lactamase (ESBL) genes that are located on mobile genetic elements [158]. It is therefore possible that cephalosporin resistance is transferred to pathogens in the digestive system of humans. This has already been demonstrated *in vitro* [159]. Dutch studies have provided indirect evidence of the foodborne transfer of ESBL genes of poultry to humans. Thirty five percent of the tested human isolates contained ESBL genes and 19% of them contained genes that were genetically identical to genes isolated from chicken meat. Eighty six percent of them contained genes that were predominant in 78% and 75% of the isolates of respectively poultry and poultry meat. Ninety four percent of the tested chicken meat isolates contained ESBL genes and 39% of them belonged to genotypes of *E. coli* that are also found in human isolates [160].

## 6. Discussion and Conclusions

The use of antibiotics in primary agricultural production is considered as an important cause of antimicrobial resistance selection in bacteria that may subsequently be found on foodstuffs. Limitation of the bacterial contamination of the primary plant and animal food products can be achieved by adherence to good agricultural practices (GAP).

*Salmonella* and *Campylobacter* are the most common causes of bacterial foodborne diseases in industrialized countries and an increasing prevalence of antimicrobial drug resistance has been recognized [152,161]. Studies have shown that infections with antimicrobial resistant *Salmonella* and *Campylobacter* can result in higher mortality as compared to infections with susceptible strains [150,151,152,153]. Therefore, special attention has to be given to reduce the prevalence of these pathogens on food products and to reduce the presence of antimicrobial resistance genes in these strains. Also, the antimicrobial resistance of zoonotic pathogens, including those that confer a risk by direct contact with living animals throughout the food chain as seen for e.g., livestock-associated methicillin resistant *Staphylococcus aureus* (LA-MRSA), have to be reduced.

Antimicrobial resistance genes present in commensal bacteria can be transferred to human pathogenic bacteria during food processing or after ingestion. Therefore it is not only recommended to monitor and reduce the presence of antimicrobial resistance genes in commensal bacteria originating from food producing animals and food products but also to study the mobile genetic elements in order to better understand their epidemiology so eventual measures can be implemented to monitor and reduce their presence in food.

Microorganisms intentionally added to foodstuffs may contain antimicrobial resistance genes and may transfer them to (pathogenic) bacteria. Also, some probiotics have the capacity to adhere to epithelial cells of the human gastrointestinal tract. Moreover, they are often ingested on a large scale. Therefore, probiotics can colonize the gastrointestinal tract for a long period of time, thereby increasing the risk of transfer of antibiotic resistance genes.

Bacteriophages can be used as agents to control foodborne bacterial pathogens and spoilage organisms. However, to perform a proper risk assessment for their use as biopreservatives in food, further research is needed with regard to the transduction potential of phages and the molecular mechanisms of the transduction process.

The effect of food processing and preservation techniques on the presence of bacteria is variable but in general the number of bacteria on foodstuffs is reduced when those techniques are applied. Conjugation is the most important way of HGT. Dead bacteria are no longer able to perform conjugation. Heat treatments reduce the risk of antimicrobial resistance genes transfer to bacteria contained in food and/or the human digestive system. Raw consumed products have the highest risk of antimicrobial resistance transfer since possibly present antimicrobial resistant bacteria are not killed by any treatment. Minimum processing and preservation techniques result in stressed bacteria, thus possibly increasing the probability of antimicrobial resistance transfer by means of conjugation [122,125]. Although minimal food processing is increasingly used in food processing, literature does not supply yet much information on the effects of these processing methods on the risk of transfer of resistance genes or resistant bacteria.

Biofilm formation is an important phenomenon in the food process. Synergistic effects between biofilm formation and plasmid transfer by conjugation have been observed which could be important in relation to the transfer of antibiotic resistance genes. In this respect, cross-resistance and co-selection between biocide resistance and antimicrobial resistance are also important.

Compliance with good manufacturing practices (GMP) and good hygienic practices (GHP) is indispensable to achieve safe food production. To limit the transfer of antibiotic resistance genes during food processing, physical parameters (such as time/temperature combinations in heat treatments) should be observed and good hygienic practices should be applied at all stages of the food chain, from farm to fork.

Finally, in the view of the increasing occurrence of antibiotic resistance in primary and processed food products, it is of the utmost importance to continue research on the quantification of the HGT of antimicrobial resistance genes to pathogens and to humans through food as well as on the correlation between virulence properties and antimicrobial resistance.
